# Cross-Section of Neurological Manifestations Among SARS-CoV-2 Omicron Subvariants—Single-Center Study

**DOI:** 10.3390/brainsci14111161

**Published:** 2024-11-20

**Authors:** Justyna Jachman-Kapułka, Aleksander Zińczuk, Krzysztof Simon, Marta Rorat

**Affiliations:** 1 6th Department of Internal Medicine, J. Gromkowski Specialist Regional Hospital, 51-149 Wroclaw, Poland; 2 1st Department of Infectious Diseases, J. Gromkowski Specialist Regional Hospital, 51-149 Wroclaw, Poland; alek.zinczuk@gmail.com (A.Z.); krzysimon@gmail.com (K.S.); 3 Clinical Department of Infectious Diseases and Hepatology, Wroclaw Medical University, 50-369 Wroclaw, Poland; 4 Department of Social Sciences and Infectious Diseases, Medical Faculty, Wroclaw University of Science and Technology, 50-370 Wroclaw, Poland; marta.rorat@gmail.com

**Keywords:** SARS-CoV-2 variants, neurologic manifestations, elderly, COVID-19 vaccine

## Abstract

**Background/Objectives**: The Omicron variant of SARS-CoV-2 is undergoing constant mutation. New strains vary in neuropathogenicity and the neurological spectrum of disease. The aim of this study was to assess the frequency and clinical characteristics of neurological manifestations during the Omicron dominance among hospitalized patients, including the differences between three subsequent periods. **Methods**: This retrospective single-center study included 426 hospitalized adults with confirmed COVID-19 divided into three periods (O1, O2, and O3) dependent on the dominance of Omicron subvariants in Poland. Demographic and clinical data, in particular neurological manifestations, were collected and compared. **Results**: The median age of the group was 74, older in subsequent (later) periods. The number of patients with a history of previous SARS-CoV-2 infection or vaccination increased with the duration of the pandemic. The severity of COVID-19 became lower in successive periods. Neurological manifestations were observed in 55.4% of patients, and the most frequent were delirium, headache, myalgia, dizziness, cerebrovascular diseases, and encephalopathy. In subsequent periods of Omicron dominance, a higher frequency of neurological manifestations such as delirium, transient ischemic attack (TIA), and encephalopathy was observed. Headache or myalgia was related to a shorter hospitalization while delirium, cerebrovascular diseases, and ischemic stroke were linked with an increased risk of death. **Conclusions**: The Omicron variant of SARS-CoV-2 presents a wide spectrum of neurological manifestations. Although there is an improvement in the survival rate of patients with COVID-19, the frequency of neurological manifestations increases. The occurrence of delirium, cerebrovascular diseases, and ischemic stroke results in higher mortality.

## 1. Introduction

In order to adapt to new hosts and environments, many viruses have the ability to generate de novo mutations in a short period of time [1]. Severe acute respiratory syndrome coronavirus 2 (SARS-CoV-2) is a new type of human coronavirus (HCoV), and it was first reported in December 2019 [2,3]. SARS-CoV-2, which causes coronavirus disease 2019 (COVID-19), has undergone natural mutations (with a very high mutation rate: around 1 × 106 to 2 × 106 mutations per nucleotide per replication cycle) since its inception as a wild type and has spread worldwide, leading to the emergence of new variants [3,4,5]. The variants of SARS-CoV-2 with significant impact on epidemiological situation are classified by the World Health Organization (WHO) as variants of concern (VOCs) [3,6]. These VOCs, responsible for subsequent waves, are Alpha (B.1.1.7), Beta (B.1.351), Gamma (P.1), Delta (B.1.617.2), and, currently dominant globally, Omicron (B.1.1.529) [3,7]. Moreover, the last variant, which was first discovered in South Africa and Botswana in November 2021, has continued to evolve in genomic regions and has led to the identification of several sublineages or subvariants which supersede previous ones approximately every 3 months (BA.1, BA.2, BA.3, BA.4, BA.5, recombinant BA.1/BA.2, and more) [8,9,10].

Due to mutations, mainly in the spike protein (S1 and S2 subunits), the variants of SARS-CoV-2 differ in severity, transmissibility, symptoms, and immune evasion [3,9,11]. Many studies confirm that the Delta variant is more transmissible than Alpha, infects younger people, and causes the most severe course, characterized by a greater requirement for oxygen treatment, and a higher risk of admission to an intensive care unit (ICU) and mortality [3,12,13,14]. In turn, the Omicron variant is less dangerous with dominant symptoms in the upper respiratory tract, often asymptomatic, with lower mortality. However, it is more transmissible and less susceptible to vaccines [9,15,16,17]. In addition, some studies show increasing evasion of neutralizing antibodies in Omicron subvariants, as well as some clinical differences between them [9,18,19,20,21,22]. 

In addition to respiratory symptoms, various neurological manifestations are associated with SARS-CoV-2 infection, with a frequency range from 18.4% to 80% [2,23]. The neurological symptoms and consequences may concern the central nervous system (CNS), peripheral nervous system (PNS), and muscles, and can appear in acute or post-acute phases of infections [2,24]. The most common neurological manifestations of COVID-19 are thought to be dizziness, headache, fatigue, neuropsychiatric and encephalopathic conditions, anosmia, ageusia, seizures, ataxia, cerebrovascular events, including ischemic stroke and intracranial hemorrhage, polyneuropathy, Guillain–Barré syndrome (GBS), Miller Fisher syndrome, meningoencephalitis, myalgia, and rhabdomyolysis [2,24,25,26]. In addition to causing infection-related neurological symptoms, SARS-CoV-2 has neuroinvasive, neurotropic, and neurovirulence properties proven both in vitro and in vivo in animal and human models [24,27,28,29]. The neuroinvasiveness of SARS-CoV-2 is the ability of a virus to enter the PNS or CNS [30]. A virus can access the CNS through peripheral nerves and/or the hematogenous pathway. SARS-CoV-2 may enter the CNS through nerve endings of cranial nerves (especially olfactory, trigeminal, and probably facial, glossopharyngeal, and vagus nerves) and spread throughout the brain by axonal (anterograde or retrograde) transport [24,29,30]. Additionally, it can penetrate the blood–brain barrier (BBB) during viremia or can infect immune system cells, which then traverse the BBB into the brain [24,29,30,31]. The virus might infect a wide range of neuronal cells and spread from one region of the brain to another, leading to pathological/cytotoxic changes in the CNS (neurovirulence) [30,31]. SARS-CoV-2 variants differ in their neuropathogenicity; this is related to different neurological symptoms [30]. Furthermore, some differences in neuropathogenic features have also been observed between sublineages of Omicron [32]. 

Given the above, it is not surprising that differences in neurological manifestations are observed between SARS-CoV-2 variants. In most studies conducted among children, more severe neurological complications of COVID-19 are observed during the dominance of Omicron [33,34]. Among adults, some studies performed with different demographics have revealed varied distributions of neurological manifestations across the pandemic [35,36,37,38]. 

The aim of this study was to assess the frequency and clinical characteristics of neurological manifestations among COVID-19 patients hospitalized in Poland during the Omicron period, depending on the dominance of the sublineages. To the best of our knowledge, this is the first study analyzing the neurological spectrum of the Omicron variant in a Polish population and the first study on adults comparing neurological manifestations among Omicron subvariants.

## 2. Materials and Methods

### 2.1. Study Design

Data for the study were collected retrospectively from an electronic medical database. The study involved patients hospitalized in the First Department of Infectious Diseases of the Specialist Regional Hospital in Wroclaw (Lower Silesia, Poland) during the dominant period for Omicron. The available data were limited only to hospital stays, without follow-up or post-hospital periods. Only symptomatic adults (≥18 years) with SARS-CoV-2 infection confirmed based on positive results of a real-time reverse transcriptase–polymerase chain reaction (RT-PCR) or rapid antigen test were included. Doubtful cases were not taken for analysis. After anonymization, a total of 426 consecutive patients were used for data analysis. The patients were divided into three groups according to the dominance of omicron subvariants:

1. Omicron period (O1) from January to June 2022—203 patients;
2. Omicron period (O2) from July 2022 to February 2023—136 patients;
3. Omicron period (O3) from March to December 2023—87 patients.

**Figure 1:**
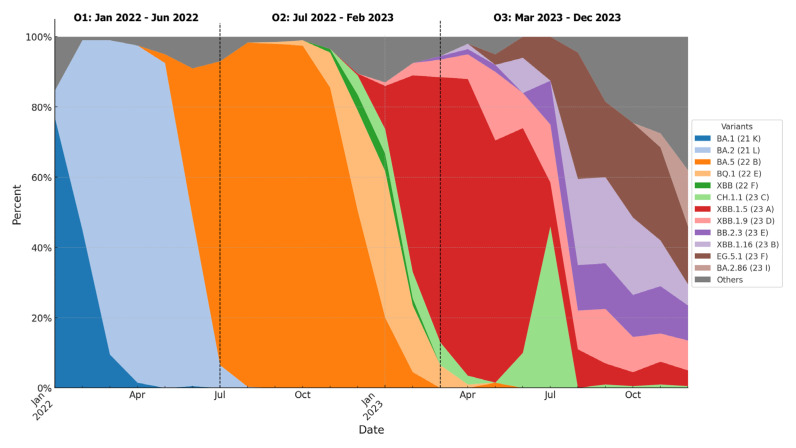
The dominance of Omicron subvariants in Poland divided into three periods. The data and graph are adapted from Nexstrain.org [39].

The division was due to the dominance of Omicron subvariants in Poland, according to the Global Initiative on Sharing All Influenza Data (GISAID). The first period was dominated by BA.1 (21 K) and BA.2 (21 L), the second by BA.5 (22 B), BQ.1 (22 E), and XBB (22 F), and the third by XBB.1.5 (23 A), CH.1.1 (23 C), XBB.1.9 (23 D), XBB.1.16 (23 B), EG.5.1 (23 F), BB.2.3 (23 E), and BA.2.86 (23 I) (Figure 1) [39]. There were no rehospitalizations due to COVID-19 during the analyzed periods.

The following data were collected: age, sex, comorbidities, COVID-19-related symptoms, duration of symptoms before admission, duration of hospitalization, SARS-CoV-2 vaccination status, previous SARS-CoV-2 infection, respiratory support, treatment, bacterial coinfections, intensive care unit (ICU) admission, and death. 

Moreover, we collected neurological manifestations (confirmed by physicians and additional tests) and divided these into neurological symptoms (headache, dizziness, myalgia, smell, taste and vision disorders, delirium, mood, memory and sleep disorders, catatonia, paresthesia, and paresis) and neurological complications (cerebrovascular diseases such as transient ischemic attack [TIA], ischemic stroke, hemorrhagic stroke and venous thrombosis, encephalopathy, seizure, ataxia, myoclonus, myopathy, mononeuropathy, polyneuropathy, GBS, meningitis, encephalitis, and myelitis). 

All collected data were compared across the three periods of Omicron dominance.

### 2.2. Statistical Analysis

The obtained results were subjected to statistical analysis. The relationship of qualitative variables was assessed using the Chi-square test. The normality of the distribution of quantitative variables in the studied groups was checked using the Shapiro–Wilk normality test. The Kruskal–Wallis test was used to examine differences between groups, because none of the analyzed quantitative variables had a normal distribution. Comparisons between groups were made using Tukey’s RIR post hoc test. The trend of changes in the case of quantitative variables was assessed using the Spearman rank correlation coefficient. A *p*-value of less than 0.05 was considered statistically significant in all analyses. The analysis was performed in Statistica 9.1 (StatSoft, Krakow, Poland) and PQStat 1.8.2.

## 3. Results

### 3.1. Overall Characteristics

**Figure 2:**
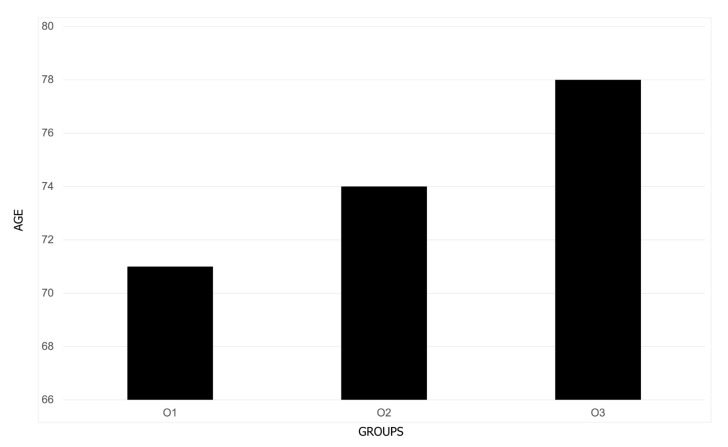
Age of patients (years; median) in three periods of Omicron dominance.

A total of 426 cases, aged 18–103 (median 74 years), 214 of which were men (50.23%), were enrolled in the study (Table 1). Patients were significantly younger during O1 than O3 (71 vs. 78 years) (Figure 2). 

The sex distribution was equal in the whole group (male *n* = 214), as well as in the following periods. The duration of hospitalization was significantly shorter in the second period than in the first period (median 5 days vs. 8 days). A total of 172/276 (62.32%) patients had been vaccinated against SARS-CoV-2 with at least one dose. The percentage of vaccinated cases increased with the duration of the pandemic (O1 52.35% < O2 76.32% < O3 83.33%). Vaccinated patients experienced a shorter time of hospitalization (median 6 days vs. 7.5 days); however, there was no statistical difference in comparison to the non-vaccinated in terms of the numbers of deaths and ICU admissions (*p* = 0.369, *p* = 0.815, respectively). In terms of clinical symptoms, delirium occurred in 24.04% of non-vaccinated and only in 8.72% of vaccinated patients (*p* < 0.001). A total of 21/405 (5.19%) patients had had a previous SARS-CoV-2 infection, with an increasing trend in each subsequent period (*p* = 0.03). Previous history of COVID-19 did not influence the occurrence of neurological manifestations or the rate of death and ICU admission. The incidence of comorbidities such as cardiovascular diseases, hypertension, past stroke/TIA, dementia, and chronic kidney disease increased during the pandemic (*p* = 0.02, *p* = 0.05, *p* < 0.001, *p* = 0.002, *p* < 0.001, respectively). Demographic characteristics used during the analysis of patients are presented in Table 1.

**Figure 3:**
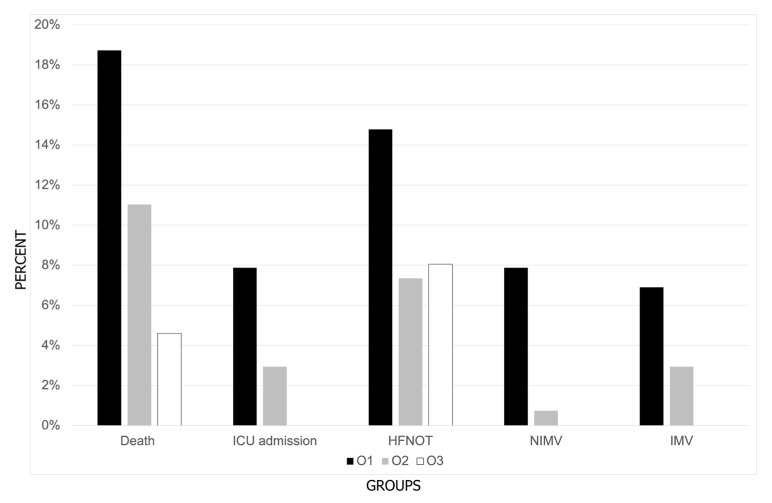
Percentage distribution of deaths, ICU admissions, and oxygen therapy among patients hospitalized with COVID-19 across three periods of Omicron dominance.

Amongst typical symptoms of COVID-19 in the subsequent periods, cough, runny nose, and weakness were reported more frequently, and dyspnea less frequently (Table 2). Remdesivir (RDV) was predominantly used in O2. A significant trend towards more frequent prescription of drugs was observed for paxlovid (PAX) and antibiotics, while the remaining drugs against SARS-CoV-2 were used less frequently in each subsequent period. Mortality, the requirement for ICU admission, as well as use of low-dose oxygen therapy, high-flow nasal oxygen therapy (HFNOT), noninvasive mechanical ventilation (NIMV), and invasive mechanical ventilation (IMV) were lower in every subsequent period of Omicron (Figure 3). Table 2 shows the characteristics of SARS-CoV-2 infection (symptoms, treatment, and outcome) among hospitalized patients. 

### 3.2. Neurological Manifestations

Neurological manifestations occurred in 55.4% of patients (n = 236); the most frequent were delirium(19.01%), headache (14.32%), myalgia (10.56%), dizziness (9.62%), cerebrovascular diseases (6.34%), and encephalopathy (6.1%), (Table 3).

**Figure 4:**
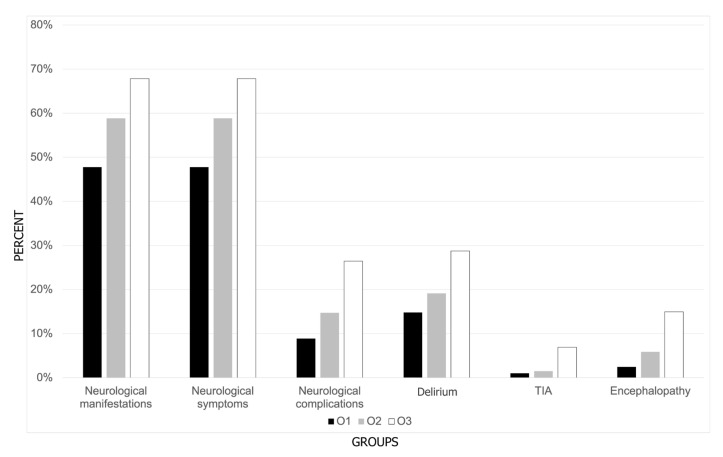
Distribution of the frequency of neurological manifestations among patients hospitalized with COVID-19 across three periods of Omicron dominance.

Over time, a higher incidence of neurological manifestations between subsequent periods was observed (O1 vs. O3 *p* = 0.005) in general, as well as in the occurrence of any kind of neurological symptoms (O1 vs. O3 *p* = 0.017) and complications (O1 vs. O3 *p* < 0.001). Furthermore, over time a higher incidence of delirium (O1 vs. O3 *p* = 0.016), TIA (O1 vs. O3 *p* = 0.015), and encephalopathy (O1 vs. O3 *p* < 0.001) were observed (Figure 4).

The correlation between the course of COVID-19 and concomitant neurological manifestations was studied. The time of hospitalization was statistically shorter when headache or myalgia were reported (median 5 days vs. 7 days), but longer when delirium occurred (median 9 days vs. 6 days). A higher risk of death, but not ICU admission, occurred in patients with neurological manifestations (19.07% vs. 6.32%; *p* < 0.001), delirium (39.51% vs. 7.25%; *p* < 0.001), cerebrovascular diseases (29.63% vs. 12.28%; *p* = 0.023), and ischemic stroke (45.45% vs. 12.53%; *p* = 0.007).

## 4. Discussion

Since the onset of COVID-19, neurological manifestations of the disease have been a common topic of many studies. Our study summarizes the neurological spectrum of SARS-CoV-2 infection during the dominance of the Omicron variant in hospitalized patients, with particular emphasis on the differences between subvariants. Over half of the analyzed patients presented at least one neurological manifestation; in China during Omicron dominance, this was one-third [40] to approximately half of patients [41]. In our research, delirium, headache, myalgia, dizziness, cerebrovascular diseases, and encephalopathy were the most frequent symptoms, whereas Shen et al.’s (2023) study was dominated by mild symptoms such as fatigue, myalgia, headache, and dizziness, with the rare presence (in comparison to our study) of cerebrovascular diseases (0.9% vs. 6.34%) [41]. 

As was mentioned in the introduction, variants of SARS-CoV-2 differ in their neuropathogenicity. For example, compared with Delta, Omicron BA.1 has reduced transmission via the olfactory nerve, with lower levels of virus replication in the olfactory mucosa [30]. Delta exhibits higher viral replication in the brain and increases neuropathology more than Omicron [32,42,43]. Furthermore, there are differential effects of SARS-CoV-2 variants on BBB (increased BBB permeability caused by wild type and Omicron) and cytopathic effects on the CNS [44]. Due to their numerous mutations and therefore different features, variants of SARS-CoV-2 have a varying incidence and spectrum of neurological manifestations. A study from India emphasized the dominance of encephalopathy, ischemic strokes, and seizures during the Omicron wave [37]. In a child population, the researchers reported an increase in severe neurological symptoms, such as seizures, alterations in consciousness, encephalopathy, and even fulminant and lethal encephalitis, throughout the Omicron period [33,34,45,46]. The latest Polish study comparing neurological manifestations across pandemic waves showed that neurological complications in general were more frequent in the pre-Delta and Omicron periods of the pandemic. The Omicron variant caused a lower percentage of smell and taste disturbances (STDs); a tendency to a higher rate of cerebrovascular diseases was observed in the pre-Delta period [38]. 

Many studies show differences in the cross-section of neurological manifestations between SARS-CoV-2 variants; however, to the best of our knowledge, there are no studies considering differences in the neurological spectrum between Omicron subvariants. In our research, the neurological manifestations (both symptoms and complications), especially delirium, TIA, and encephalopathy, occurred more frequently in subsequent periods of Omicron dominance. It is known that Omicron, compared with the previous VOCs, increases BBB permeability and may lead to more severe neurological damage through its ability to induce neuronal stress, affect extracellular glutamate concentrations, and damage BBB cells [24,30,32]. There are insufficient studies analyzing the differing neuropathogenicity between subvariants of Omicron. One research project on a mice model by Wang et al. (2023) found that BA.5.2 causes stronger immune suppression and neurodegeneration, while BA.2.75 exhibits more similar characteristics to Delta in the cortex, which may exacerbate neurological sequelae by BA.5.2 [32]. An additional risk factor for neurological complications in our study is older age. 

Despite the more frequent neurological manifestations in subsequent periods of Omicron in the analyzed cohort, the general course of the disease became increasingly less severe. Each successive group was older with more comorbidities, but also with a history of vaccinations against SARS-CoV-2. In the subsequent periods, symptoms of COVID-19 came from the upper respiratory tract. The later Omicron cases had a reduced requirement for oxygen therapy and mechanical ventilation, and thus lower mortality and necessity for admission to ICU. Our results are similar to the Polish study by Flisiak et al. (2023) which revealed that during the later period of Omicron dominance (BA.5, BQ.1, XBB, XBB.1.5, XBB.1.9) hospitalized patients were older, albeit with a less severe course and better prognosis in comparison to early periods (BA.1 and BA.2) [22]. Moreover, an American study emphasized that during SARS-CoV-2 infection from the Delta-predominant period to the post-BA.4/BA.5 period, the proportion of ICU admissions, in-hospital mortality, use of NIMV and IMV, and duration of hospitalization decreased [47]. Nevertheless, the literature shows that advanced age and comorbidities such as cancer, hypertension, and diabetes mellitus are considered as the independent risk factors of death among COVID-19 patients [48,49]. In our research, despite there being older patients with a greater percentage of cardiovascular diseases, past stroke/TIA, dementia, and chronic kidney disease over time, the course of infection became less severe, with a lower mortality rate. This may indicate that changes in Omicron’s genetic material are crucial for the severity of the disease. Although prior vaccination against SARS-CoV-2 had an impact on the shorter time of hospitalization and lower rate of altered mentation, there was no correlation between vaccination status and mortality or ICU admission. A study from Ireland proved that during the period of Omicron dominance vaccination reduced morbidity and mortality in the population [50]. However, the Omicron variant has a high immune escape capacity and some vaccine escape ability, which could explain the lower efficiency of vaccines in our cohort [9,51]. Our vaccinated group was quite small (n = 172) and patients received different amounts of vaccine dose at different times. This could falsify the conclusions that the vaccine against SARS-CoV-2 did not affect the course of COVID-19.

Neurological risk and prognostic factors of the severity of SARS-CoV-2 infection are often highlighted in many studies. In this research, the occurrence of any neurological manifestation, delirium, cerebrovascular diseases, and ischemic stroke was associated with a higher mortality rate, which confirmed the results from other studies in Poland [38,52]. Moreover, studies conducted on various demographic groups have shown that a decreased level of consciousness, delirium, and hemorrhagic or ischemic stroke comorbid with COVID-19 increase the risk of in-hospital mortality [47,53,54,55,56]. In particular, delirium (with a frequency range from 6 to 67% from the literature) is one of the strongest predictors of poor outcome in COVID-19 patients [55,57,58]. In our study, delirium was the most common neurological symptom (19.01%), which is similar to an Italian study [57] describing delirium affecting 22% of hospitalized patients with COVID-19. This study by Di Giorgio et al. [57] emphasizes delirium as the strongest independent predictor of death/admission to ICU. Furthermore, the same study shows that several clinical features (such as dementia or age) and inflammatory markers are predictors of delirium development [57]. In our research, delirium extended hospitalization time and, in addition, was the most significant factor of death occurrence. The mortality rate of COVID-19 patients with delirium was over five times higher than the mortality rate of those without delirium, which is an even worse result than in the study by Wu et al. [59], where the mortality rate in patients with delirium was more than twice as high.

In our study, the occurrence of headache or myalgia shortened the hospitalization time. It has been confirmed by many studies that the presence of headache during COVID-19 is a good predictor of infection course and decreases the risk of in-hospital mortality [41,52,60,61,62]. 

All these results indicate the importance of following neurological complications during COVID-19, since their presence may have an impact on the severity of SARS-CoV-2 infection. 

There are some limitations of this research. Firstly, this is a retrospective study conducted in a single medical center with hospitalized patients, which has resulted in a limited group size. Secondly, there are gaps in medical records, especially in additional tests, which may result in a lower number of diagnosed neurological complications. Thirdly, due to the lack of genetic verification of the virus subvariant, the division was made on the basis of data provided by GISAID.

## 5. Conclusions

Neurological manifestations during infection with the Omicron variant of SARS-CoV-2 occur in more than half of hospitalized patients. The frequency increased over the duration of the pandemic and subsequent mutations. The occurrence of altered mentation, cerebrovascular diseases, and ischemic stroke during Omicron is a risk factor of death. Nevertheless, the course of COVID-19 becomes less severe in subsequent subvariants with lower mortality and ICU admission rates. Given the differences in the neurological spectrum between Omicron subvariants, and the Omicron variant’s constant mutation, further studies on the neuropathogenicity and clinical differences of virus sublineages should be conducted.

## Figures and Tables

**Table 1:** Demographic characteristics of patients hospitalized with COVID-19 during the period of Omicron dominance in Poland. Comparison of the data between three periods. Quantitative parameters and variables are presented as mean values (standard deviation—SD) and medians [interquartile range—IQR] and qualitative variables are presented as frequency/number.

Variables	Total (*n* = 426)	O1 (*n* = 203)	O2 (*n* = 136)	O3 (*n* = 87)	*p*-Value	Trend *p*-Value
Age (years)	71.69 (17.55) 74.00 [65–85]	68.28 (19.23) 71.00 [60–83]	73.27 (15.34) 74.00 [67–85]	77.21 (14.87) 78.00 [71–88]	*p* < 0.001	*p* < 0.001
Sex						
Male	214	104 (51.23%)	68 (50.00%)	42 (48.28%)	*p* = 0.9	*p* = 0.643
Female	212	99 (48.77%)	68 (50.00%)	45 (51.72%)		
Duration of symptoms before admission (days)	4.75 (4.60) 3.00 [2–7]	5.30 (4.85) 4 [2–7]	4.13 (4.41) 3 [1–5]	4.46 (4.21) 3 [1–7]	*p* = 0.109	*p* = 0.03
Length of hospitalization (days)	9.00 (10.40) 7.00 [5–0]	9.99 (8.59) 8.00 [5–12]	8.47 (14.72) 5 [4–8]	7.53 (3.98) 7 [5–10]	*p* = 0.004	*p* = 0.006
SARS-CoV-2 vaccination (≥1 dose)	172 (62.32%) (n = 276)	89 (52.35%) (n = 170)	58 (76.32%) (n = 76)	25 (83.33%) (n = 30)	*p* < 0.001	*p* < 0.001
Previous SARS-CoV-2 infection	21 (5.19%) (n = 405)	7 (3.47%) (n = 202)	7 (5.15%) (n = 136)	7 (10.45%) (n = 67)	*p* = 0.08	*p* = 0.04
Comorbidities						
Cardiovascular diseases	324 (76.06%)	146 (71.92%)	105 (77.21%)	73 (83.91%)	*p* = 0.084	*p* = 0.026
Hypertension	280 (65.73%)	125 (61.58%)	91 (66.91%)	64 (73.56%)	*p* = 0.135	*p* = 0.046
Atrial fibrillation	21 (4.93%)	9 (4.43%)	7 (5.15%)	5 (5.75%)	*p* = 0.885	*p* = 0.621
Ischemic heart disease	16 (3.76%)	7 (3.45%)	6 (4.41%)	3 (3.45%)	*p* = 0.888	*p* = 0.907
Chronic circulatory failure	7 (1.64%)	5 (2.46%)	1 (0.74%)	1 (1.15%)	*p* = 0.434	*p* = 0.306
Diabetes	114 (26.76%)	54 (26.60%)	31 (22.79%)	29 (33.33%)	*p* = 0.222	*p* = 0.396
Respiratory diseases	63 (14.79%)	30 (14.78%)	20 (14.71%)	13 (14.94%)	*p* = 0.999	*p* = 0.978
Cancer history	82 (19.25%)	48 (23.65%)	18 (13.24%)	16 (18.39%)	*p* = 0.057	*p* = 0.127
Chronic kidney disease	41 (9.62%)	12 (5.91%)	11 (8.09%)	18 (20.69%)	*p* < 0.001	*p* < 0.001
Obesity	42 (9.86%)	27 (13.30%)	8 (5.88%)	7 (8.05%)	*p* = 0.066	*p* = 0.074
Neurological comorbidities						
Past history of stroke/TIA	63 (14.79%)	15 (7.39%)	29 (21.32%)	19 (21.84%)	*p* < 0.001	*p* < 0.001
Dementia	69 (16.20%)	23 (11.33%)	23 (16.91%)	23 (26.44%)	*p* = 0.006	*p* = 0.002
Epilepsy	17 (3.99%)	6 (2.96%)	8 (5.88%)	3 (3.45%)	*p* = 0.386	*p* = 0.605
Parkinson’s disease	17 (3.99%	6 (2.96%)	5 (3.68%)	6 (6.90%)	*p* = 0.283	*p* = 0.141
Multiple sclerosis	4 (0.939%)	2 (0.99%)	2 (1.47%)	0 (0.00%)	*p* = 0.537	*p* = 0.557
Neuromuscular disease	6 (1,41%)	3 (1.48%)	2 (1.47%)	1 (1.15%)	*p* = 0.974	*p* = 0.847
CNS Neoplasm	7 (1,64%)	4 (1.97%)	3 (2.21%)	0 (0.00%)	*p* = 0.399	*p* = 0.309

Abbreviations: O1: first period of Omicron dominance; O2: second period of Omicron dominance; O3: third period of Omicron dominance; TIA: transient ischemic attack; CNS: central nervous system.

**Table 2:** Characteristics of SARS-CoV-2 infection among patients hospitalized with COVID-19 during the period of Omicron dominance in Poland. Comparison of the data between three periods. Qualitative variables are presented as frequency/number.

Variables	Total (*n* = 426)	O1 (*n* = 203)	O2 (*n* = 136)	O3 (*n* = 87)	*p*-Value	Trend *p*-Value
Systemic features						
Fever	236 (55.40%)	110 (54.19%)	70 (51.47%)	56 (64.37%)	*p* = 0.149	*p* = 0.199
Cough	210 (49.30%)	90 (44.33%)	68 (50.00%)	52 (59.77%)	*p* = 0.054	*p* = 0.017
Dyspnea	160 (37.56%)	92 (45.32%)	39 (28.68%)	29 (33.33%)	*p* = 0.005	*p* = 0.013
Diarrhea	46 (10.80%)	24 (11.82%)	10 (7.35%)	12 (13.79%)	*p* = 0.258	*p* = 0.916
Runny nose	45 (10.56%)	15 (7.39%)	16 (11.76%)	14 (16.09%)	*p* = 0.075	*p* = 0.023
Sore throat	57 (13.38%)	23 (11.33%)	24 (17.65%)	10 (11.49%)	*p* = 0.208	*p* = 0.645
Rash	8 (1.88%)	6 (2.96%)	1 (0.74%)	1 (1.15%)	*p* = 0.287	*p* = 0.196
Fatigue	274 (64.32%)	118 (58.13%)	92 (67.65%)	64 (73.56%)	*p* = 0.026	*p* = 0.007
Loss of appetite	79 (18.54%)	35 (17.24%)	26 (19.12%)	18 (20.69%)	*p* = 0.770	*p* = 0.043
Respiratory support						
No oxygen	180 (42.25%)	74 (36.45%)	65 (47.79%)	41 (47.13%)	*p* = 0.069	*p* = 0.044
Low-dose oxygen therapy	246 (57.75%)	129 (63.55%)	71 (52.21%)	46 (52.875)	*p* = 0.069	*p* = 0.044
HFNOT	47 (11.03%)	30 (14.78%)	10 (7.35%)	7 (8.05%)	*p* = 0.062	*p* = 0.043
NIMV	17 (3.99%)	16 (7.88%)	1 (0.74%)	0 (0.00%)	*p* < 0.001	*p* < 0.001
IMV	18 (4.26%)	14 (6.90%)	4 (2.94%)	0 (0.00%)	*p* = 0.019	*p* = 0.005
Treatment						
Dexamethasone	189 (44.37%)	113 (55.67%)	41 (30.15%)	35 (40.23%)	*p* < 0.001	*p* < 0.001
Chloroquine/hydroxychloroquine	1 (0.23%)	1 (0.49%)	0 (0.00%)	0 (0.00%)	*p* = 0.577	*p* = 0.350
Amantadine	2 (0.47%)	1 (0.49%)	0 (0.00%)	1 (1.15%)	*p* = 0.472	*p* = 0.622
COVID-19 convalescent plasma	1 (0.23%)	1 (0.49%)	0 (0.00%)	0 (0.00%)	*p* = 0.577	*p* = 0.350
Paxlovid	21 (4.23%)	0 (0.00%)	6 (4.41%)	15 (17.24%)	*p* < 0.001	*p* < 0.001
Antibodies (casiriwimab/imdewimab or regdanvimab)	3 (0.70%)	3 (1.48%)	0 (0.00%)	0 (0.00%)	*p* = 0.190	*p* = 0.104
Remdesivir	181 (42.49%)	76 (37.44%)	91 (66.91%)	14 (16.09%)	*p* < 0.001	*p* = 0.110
Tocilizumab	21 (4.23%)	18 (8.87%)	2 (1.47%)	1 (1.15%)	*p* = 0.002	*p* = 0.001
Baricitinib	10 (2.35%)	10 (4.93%)	0 (0.00%)	0 (0.00%)	*p* = 0.004	*p* = 0.003
Molnupiravir	26 (6.10%)	12 (5.91%)	11 (8.09%)	3 (3.45%)	*p* = 0.365	*p* = 0.618
Anticoagulation p.o. (VKA, NOAC)	32 (7.51%)	12 (5.91%)	5 (3.68%)	15 (17.24%)	*p* < 0.001	*p* = 0.006
LMWH	383 (89.91%)	184 (90.64%)	129 (94.85%)	70 (80.46%)	*p* = 0.002	*p* = 0.045
Antibiotics	175 (41.08%)	102 (50.25%)	42 (30.88%)	31 (35.63%)	*p* < 0.001	*p* = 0.003
Bacterial coinfections	104 (24.41)	58 (28.57)	26 (19.12%)	20 (22.99%)	*p* = 0.132	*p* = 0.156
ICU admission	20 (4.69%)	16 (7.88%)	4 (2.94%)	0 (0.00%)	*p* = 0.007	*p* = 0.002
Death	57 (13.38%)	38 (18.72%)	15 (11.03%)	4 (4.60%)	*p* = 0.003	*p* < 0.001

Abbreviations: O1: first period of Omicron dominance; O2: second period of Omicron dominance; O3: third period of Omicron dominance; HFNOT: high-flow nasal oxygen therapy; NIMV: noninvasive mechanical ventilation; IMV: invasive mechanical ventilation; p.o.: per os; VKA: vitamin K antagonists; NOAC: non-vitamin K antagonist oral anticoagulants; LMWH: low-molecular-weight heparin; ICU: intensive care unit.

**Table 3:** Neurological manifestations (symptoms and complications) in patients hospitalized with COVID-19 across three periods of Omicron dominance in Poland.

Variables	Total (n = 426)	O1 (n = 203)	O2 (n = 136)	O3 (n = 87)	*p*-Value	Trend *p*-Value
Neurological manifestations	236 (55.40%)	97 (47.78%)	80 (58.82%)	59 (67.82%)	*p* = 0.004	*p* = 0.001
Neurological symptoms	236 (55.40%)	97 (47.78%)	80 (58.82%)	59 (67.82%)	*p* = 0.011	*p* = 0.003
Headache	61 (14.32%)	24 (11.82%)	26 (19.12%)	11 (12.64%)	*p* = 0.151	*p* = 0.521
Dizziness	41 (9.62%)	18 (8.87%)	12 (8.82%)	11 (12.64%)	*p* = 0.564	*p* = 0.380
Myalgia	45 (10.56%)	17 (8.37%)	17 (12.50%)	11 (12.64%)	*p* = 0.374	*p* = 0.206
Smell disorder	6 (1.41%)	3 (1.48%)	1 (0.74%)	2 (2.30%)	*p* = 0.623	*p* = 0.738
Taste disorder	6 (1.41%)	3 (1.48%)	1 (0.74%)	2 (2.30%)	*p* = 0.623	*p* = 0.738
Vision disorder	2 (0.47%)	0 (0.00%)	1 (0.74%)	1 (1.15%)	*p* = 0.363	*p* = 0.160
Delirium	81 (19.01%)	30 (14.78%)	26 (19.12%)	25 (28.74%)	*p* = 0.021	*p* = 0.007
Mood disorder	35 (8.22%)	15 (7.39%)	14 (10.29%)	6 (6.90%)	*p* = 0.559	*p* = 0.904
Catatonia	6 (1.41%)	0 (0.00%)	3 (2.21%)	3 (3.45%)	*p* = 0.047	*p* = 0.014
Memory disorder	24 (5.63%)	10 (4.93%)	8 (5.88%)	6 (6.90%)	*p* = 0.780	*p* = 0.481
Sleep disorder	16 (3.76%)	10 (4.93%)	6 (4.41%)	0 (0.00%)	*p* = 0.115	*p* = 0.065
Paresthesia	8 (1.88%)	4 (1.97%)	3 (2.21%)	1 (1.15%)	*p* = 0.844	*p* = 0.707
Paresis	27 (6.34%)	8 (3.94%)	16 (11.76%)	3 (3.45%)	*p* = 0.007	*p* = 0.534
Neurological complications	61 (14.32%)	18 (8.87%)	20 (14.71%)	23 (26.44%)	*p* < 0.001	*p* < 0.001
Cerebrovascular diseases	27 (6.34%)	9 (4.43%)	10 (7.35%)	8 (9.20%)	*p* = 0.263	*p* = 0.105
TIA	10 (2.35%)	2 (0.99%)	2 (1.47%)	6 (6.90%)	*p* = 0.007	*p* = 0.006
Ischemic stroke	11 (2.58%)	4 (1.97%)	5 (3.68%)	2 (2.30%)	*p* = 0.613	*p* = 0.696
Hemorrhagic stroke	1 (0.23%)	1 (0.49%)	0 (0.00%)	0 (0.00%)	*p* = 0.577	*p* = 0.350
Venous thrombosis	5 (1.17%)	2 (0.99%)	3 (2.21%)	0 (0.00%)	*p* = 0.310	*p* = 0.712
Encephalopathy	26 (6.10%)	5 (2.46%)	8 (5.88%)	13 (14.94%)	*p* < 0.001	*p* < 0.001
Seizure	7 (1.64%)	3 (1.48%)	2 (1.47%)	2 (2.30%)	*p* = 0.865	*p* = 0.657
Ataxia	1 (0.23%)	1 (0.49%)	0 (0.00%)	0 (0.00%)	*p* = 0.577	*p* = 0.350
Myoclonus	1 (0.23%)	0 (0.00%)	0 (0.00%)	1 (1.15%)	*p* = 0.142	*p* = 0.102
Myopathy	1 (0.23%)	0 (0.00%)	0 (0.00%)	1 (1.15%)	*p* = 0.142	*p* = 0.102
Mononeuropathy	1 (0.23%)	1 (0.49%)	0 (0.00%)	0 (0.00%)	*p* = 0.577	*p* = 0.350
Polyneuropathy	3 (0.70%)	1 (0.49%)	2 (1.47%)	0 (0.00%)	*p* = 0.389	*p* = 0.892
GBS	1 (0.23%)	1 (0.49%)	0 (0.00%)	0 (0.00%)	*p* = 0.577	*p* = 0.350
Meningitidis	2 (0.47%)	2 (0.99%)	0 (0.00%)	0 (0.00%)	*p* = 0.333	*p* = 0.186
Encephalitis	1 (0.23%)	1 (0.49%)	0 (0.00%)	0 (0.00%)	*p* = 0.577	*p* = 0.350
Myelitis	1 (0.23%)	1 (0.49%)	0 (0.00%)	0 (0.00%)	*p* = 0.577	*p* = 0.350

Abbreviations: O1: first period of Omicron dominance; O2: second period of Omicron dominance; O3: third period of Omicron dominance; TIA: transient ischemic attack; GBS: Guillain–Barré syndrome.

## Data Availability

All relevant data are within the manuscript.
